# The ties that bind: Cradling in Tajikistan

**DOI:** 10.1371/journal.pone.0204428

**Published:** 2018-10-31

**Authors:** Lana B. Karasik, Catherine S. Tamis-LeMonda, Ori Ossmy, Karen E. Adolph

**Affiliations:** 1 Department of Psychology, College of Staten Island & Graduate Center, CUNY, Staten Island, New York, United States of America; 2 Department of Applied Psychology, New York University, New York, New York, United States of America; 3 Department of Psychology, New York University, New York, New York, United States of America; TNO, NETHERLANDS

## Abstract

A traditional childrearing practice—“gahvora” cradling—in Tajikistan and other parts of Central Asia purportedly restricts movement of infants’ body and limbs. However, the practice has been documented only informally in anecdotal reports. Thus, this study had two research questions: (1) To what extent are infants’ movements restricted in the gahvora? (2) How is time in the gahvora distributed over a 24-hour day in infants from 1–24 months of age? To answer these questions, we video-recorded 146 mothers cradling their infants and interviewed them using 24-hour time diaries to determine the distribution of time infants spent in the gahvora within a day and across age. Infants’ movements were indeed severely restricted. Although mothers showed striking uniformity in how they restricted infants’ movements, they showed large individual differences in amount and distribution of daily use. Machine learning algorithms yielded three patterns of use: day and nighttime cradling, mostly nighttime cradling, and mostly daytime cradling, suggesting multiple functions of the cradling practice. Across age, time in the gahvora decreased, yet 20% of 12- to 24-month-olds spent more than 15 hours bound in the gahvora. We discuss the challenges and benefits of cultural research, and how the discovery of new phenomena may defy Western assumptions about childrearing and development. Future work will determine whether the extent and timing of restriction impacts infants’ physical and psychological development.

## The gahvora cradle

**Fig 1:**
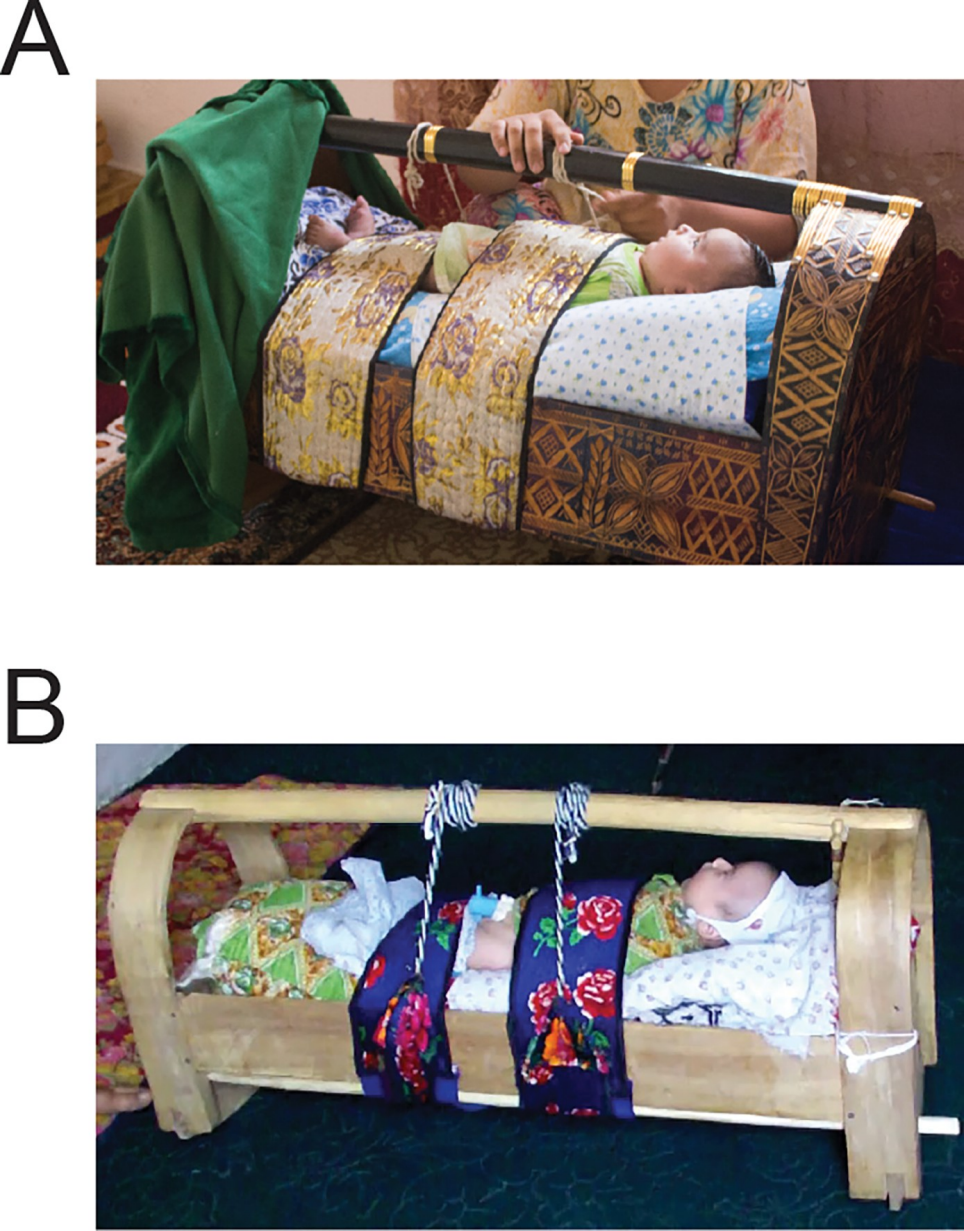
(A) Gahvora with intricately carved details, fancy bindings, and coverings. (B) Plain-looking gahvora from a less affluent household.

Several years ago, UNICEF of Tajikistan brought to our attention an unpublished report by Save the Children that described a traditional “gahvora” cradling practice in Central Asia (Fig 1). According to the report, infants from birth to 20 months of age are bound on their backs in a tightly wrapped swaddle with arms extended along the sides of the torso and legs straightened and tied together for up to 20 hours per day [1]. Thus, “the child cannot move its arms and legs, and cannot turn from side to side, only the head can be moved slightly sideways” [1]. Infants are not unwrapped for feeding because mothers lean over the cradle to breastfeed and they are not removed for toileting because infants urinate through an external catheter and defecate through a hole in the bottom of the cradle.

This description of gahvora cradling sparked our interest because of the stark contrast to historical and contemporary accounts of childrearing practices around the globe. Prior work showed that long periods of restricted movement in swaddling cloths, on cradleboards, in a manta pouch, and so on are limited to the first few months after birth and to times of day when infants are asleep [2–5]. In fact, freedom to move is a central tenet of most caregiving practices. Some cultures even promote infant movement through exercise, stretching, massage, and special devices, toys, and equipment designed to stimulate infants’ spontaneous motility, and manual, postural, and locomotor skills [2]. If verified, restriction in the gahvora cradle would expand researchers’ view of cultural differences in everyday childrearing practices.

Moreover, according to the prevailing view among pediatricians and psychologists, severe movement restriction in infancy could have deleterious effects, especially across the first two postnatal years, a critical period in children’s health and development [6]. For example, the extended and abducted position of the legs, especially in the first few months, could lead to hip dysplasia or pigeon-toed gait [5, 7]. Extended time in the supine position could lead to brachycephaly and plagiocephaly, flattening of the skull, or torticollis, a habitual asymmetry in head position [8–9]. Restricted movement—especially in older infants—could delay development of postural and locomotor skills [10]. And the claim by Save the Children that infants are fed and toileted in the cradle implies that infants have no reprieve from constraint.

To our surprise, the gahvora cradling practice is undocumented in the literature—as is the case for infant and child development in Central Asia more generally [2]. The report by Save the Children was based on anecdotal evidence from only seven mothers and an unspecified number of mothers who participated in informal focus groups. Thus, critical facts about this childrearing practice are unknown: Does gahvora cradling indeed constrain infant movement to the extent described by Save the Children? Perhaps infants’ limb and torso movements are not severely restricted. Are infants bound in the cradle for substantial hours each day? Perhaps restriction is limited only to naps and night sleep. And does the practice continue into infants’ second year when postural and locomotor skills rapidly develop [11]? Perhaps caregivers do not cradle infants once they begin to exhibit prone movements or sit up. Before investigating effects of movement restriction due to this childrearing practice, it is necessary to document the extent and timing of restriction.

## Current study

Thus, we provide the first quantitative description of the gahvora cradling practice. We aimed to document: (1) the extent to which infants’ movements are restricted in the gahvora (by video-recording mothers cradling their infants); and (2) the distribution of cradling over the course of a 24-hour day and the duration of use across development (using a time-diary method). For purposes of fidelity, transparency, and reproducibility [12], we video recorded the entire session, including consent to participate, formal instructions, informal conversations, and procedures for the cradling practice and time-diary interview. These procedural videos and the raw data are shared with the developmental research community on Databrary.org.

Our primary interest was whether infants were indeed laid supine, catheterized, and swaddled neck to toe with arms and legs extended as reported by Save the Children. In addition, we examined whether all mothers or only some engaged in the various components of cradling, and whether the components occurred in a consistent sequence across mothers. For example, in Western cultures, ointments and powder are optional components of diapering, and can be applied before or after placing the open diaper under the infant’s bottom; but wrapping the diaper around the infants’ thighs must precede fastening the diaper. Similarly, we reasoned that some mothers, not all, would catheterize their infants, and that catheterizing could occur at any point prior to draping.

Rather than rely on mothers’ self-reported measures on the extent of restriction and speculations about how much or how little they use the gahvora with their infants [i.e., 1], we opted for more precise and systematic measures of the practice. We used detailed video coding to quantify the materials, steps, and timing of the gahvora process and examined differences across infants’ age. Using time-diaries, mothers gave an hourly account of their infant’s day, creating a profile of their infant’s whereabouts and instances of restriction over the course of the day. To categorize infants based on their gahvora use, we implemented a data-driven, machine-learning approach eliminating the need to impose assumptions about daily routines based on Western norms and expectations.

## Method

### Establishing partnerships and training Tajik researchers

Our first step was to secure permission from the Ministry of Health of Tajikistan to carry out the project. We established partnerships with UNICEF and Save the Children of Tajikistan to gain access to families and collect data. The first author visited Tajikistan three times to train two Tajik researchers (a pediatrician and an assistant), observe 8 preliminary home visits, and meet informally with Tajik families. She stayed with a family overnight to observe the flow of life in a Tajik village. The Tajik researchers visited our lab in NYC for additional training. Based on this preliminary work, the authors and Tajik researchers settled on a final protocol.

After data collection commenced, the first author communicated with the Tajik researchers 1–2 times per week via video conference calls and email to prevent drift in the protocol and to clarify issues that arose as we coded and analyzed the data. Most communication occurred in Russian with the first author. Fifteen sessions were translated into Russian and English to ensure fidelity of the protocol.

### Identifying regions of study

Our second step in designing the study was to identify regions for data collections. UNICEF and Save the Children recommended that we recruit families from rural areas outside the capital city of Dushanbe, where they had contacts in village clinics and where we were likely to observe the practice in its traditional form. We focused on the warmer, arid, Khatlon district and the colder, mountainous, Rasht district. Data were collected over 16 months, from June to October, when temperatures ranged from 8°C (46°F) in the winter to 42°C (108°F) in the summer.

Villages in Khatlon and Rasht are organized in a collective arrangement. Each family lives in a single-room, one-story clay home. Several homes are clustered around a small courtyard or garden with fruits, nuts, and vegetables grown for consumption and trade. Infants are surrounded by many adults and children (3–26 people in each compound, *M* = 10 people) and cared for by parents, relatives, and neighbors. Thus, even singleton children are not “only children” as in typical U.S. families.

Chairs, tables, and beds are rare. Instead, indoor surfaces are covered in carpets and “kurpacha”—ornate, narrow, quilted mats (5 cm thick, 2 m long, 1 m wide)—that serve as seating and bedding. Kurpacha stand in tall stacks and are laid on the floor for mealtime and bedtime. Children and adults squat (buttocks to heels), kneel, or sit cross-legged on the kurpacha around a “dastarkhan,” a cloth on which food and drink are placed. Low “tapchan” platforms (3–5 m^2^)—made of metal or wood and lined with kurpacha—on the porch or in the courtyard are used for meals and sleep during warm weather.

The water system in rural homes is underdeveloped without a method of purification. Some compounds have cisterns, which collect rainwater or divert water from irrigation ditches running through the courtyard. In some compounds, water is piped to kitchens or outdoor cooking areas. The communal toilet is a hole in the ground surrounded by concrete on which older children and adults squat. Bathing also occurs in a communal space.

In addition to day/night cycles, families can monitor time in several ways. Most families have at least one clock and cell phone that show time. Most families are Muslim and pray five times a day: before dawn, midday, around 4 p.m., before sundown, after sundown, and at bedtime. Electricity is limited, and gets switched on from 5–7 a.m. and from 9–11 p.m. Typically, in the evenings when electricity is available, the family gathers to eat dinner and watch TV until electricity is turned off, and everyone goes to sleep.

### Transferring data from Tajikistan to NYC

Our third step in planning this cultural study was to devise a way to transfer data from Tajikistan to NYC. The large file size of the videos posed a practical problem: Internet is intermittent in Tajikistan, and bandwidth is limited. To surmount this problem, the Tajik researcher split the video files and other data into hundreds of smaller archived files (using 7-Zip.org) and then uploaded them onto a shared drive for transmission; a complementary program on our side recombined the files into their original form. This process took several days per file due to frequent Internet interruptions. The researcher also sent files on a hard drive through an international shipping service or used friends and relatives as couriers.

### Participants

The Tajik researchers recruited families from medical clinics based on infants’ age and mothers’ confirmation that infants were born at term without complications. Researchers informed medical personnel and mothers that the purpose of the study was to learn about infants’ daily routines and development, and did not tell them that gahvora use was our primary interest. Verbal consent was obtained from parents prior to participation; the University Integrated Institutional Review Board of the College of Staten Island, City University of New York approved this study and the verbal consent procedures. Families received infant pajamas and a toy for participation.

Over a two-year period, we collected 185 cross-sectional observations when infants were 1, 4, 8, 12, 16, 20, or 24 months of age (± 1 week). Data from 11 families could not be used because 3 mothers never cradled their infants and 8 mothers stopped using the cradle before the test date. Mothers (*n* = 168) and their infants (82 girls, 86 boys) were visited at home for 1.5 to 2 hours: 146 contributed video data on the gahvora practice, 147 contributed time diary data; 125 contributed both types of data. Data were missing from the gahvora practice because 5 caregivers misunderstood instructions, 18 infants were asleep, 3 were sick, and 2 videos were damaged. Data were missing from the time diary because 9 caregivers did not give a 24-hour account of the previous day, and 18 reported illness or other circumstances that deviated from their typical daily activities (e.g., family was travelling; family’s gahvora was being used by a visiting guest).

Families had between 1 and 8 children at the time of data collection (*M* = 2.36 children). All mothers breastfed infants from birth. Mothers ranged from 18 to 42 years of age (*M* = 26.3). Fifty-three percent of mothers had completed secondary school (11 years of education), 29% finished primary school (4 years), 11% had no education, and 7% completed more than 11 years of schooling. All mothers spoke Tajik as their primary language; a few spoke Uzbek; none spoke English. So, all sessions were conducted in Tajik. Most mothers did not work for pay (82%); 12% worked on the collective farm; and 6% did odd jobs. Most mothers (98%) were married; the rest were divorced or widowed. Forty-four percent of fathers were migrant workers in Russia and did not live at home; 28% lived at home and did odd jobs; 9% worked in construction; 7% were drivers; and 12% did not work.

### Video recordings of cradling practice

To understand the gahvora practice and to document the extent of infants’ restriction in the cradle, the researcher asked mothers to show their gahvora and put their baby into the gahvora as they normally would. The researcher video recorded the gahvora, all the materials associated with the cradle, and how they were used in the cradling process.

A primary coder used Datavyu (www.datavyu.org), a computerized software that time-locks video to user-defined behaviors with frame accuracy, to score the onset of each time that the caregiver touched one of the cradling materials. The cradling process began when the mother placed her hands on the infant to position the infant in the gahvora and ended when the mother stopped touching the cradle materials. The coder also noted whether infants were naked/clothed, breast- or bottle-fed, rocked, and whether infants fussed or resisted by pushing mothers’ hands or gahvora materials away. A second coder independently scored 33%– 100% of each infant’s session. Inter-observer reliability was high: agreement on the order (99.0%), number (99.2%), and types of materials (99.7%) used in the cradling process, and *r*s = .95–.99, *p* < .001 for the duration of each component; 99% agreement on rocking, 100% on breastfeeding, and 96.0% on fussing/resisting (κs = .78–1.0, *p* < .001).

For 10 sessions, grandmothers demonstrated the cradling procedure. Mothers and grandmothers did not differ on cradling, so data were merged for analyses. The Tajik pediatrician (who had not conducted the home visits) viewed 10 randomly selected videos of the gahvora demonstration and confirmed that mothers seemed comfortable as they cradled their infants.

### Time diaries of daily cradle use

We used a time-diary approach to document the duration and distribution of time when infants were in the gahvora throughout the previous 24-hour day when details were still fresh in mothers’ minds. Time diaries have been validated in prior work on parenting [13–15]. The researcher first confirmed whether the previous day was typical (infant not ill, family not travelling, etc.). Then mothers provided an hourly account of their infant’s whereabouts. The researcher led the mother through the 24 hours from 6 a.m. on the previous day until 6 a.m. on the test day, asking about the infant’s location over the course of the day, prompting mother with, “What happened next?” The researcher wrote mothers’ responses on a form that was gridded in 5-minute increments, creating a timeline of when infants were in and out of the gahvora. After completing the time diary, the researcher asked mothers about their infant’s age when they started using the gahvora.

The first author verified questionable timelines (e.g., baby taken out of gahvora in the middle of the night; baby on ground for full 24-hour day) by checking diaries against videos of the interviews and the researcher’s online notes with the Tajik researchers. We also verified timelines of 15 randomly selected sessions against verbatim transcripts that were translated from Tajik into Russian or English. We created new timelines from the translations and compared them with the diaries coded online (100% on location and *r* = .99, *p* < .001 duration in the gahvora). To ensure data entry accuracy, diaries were double entered and disagreements were resolved.

We had no a-priori reason to assume that Tajik families adhere to U.S.-typical wakeup and bedtimes, nap times, and meal times. So, rather than imposing assumptions about daily schedules based on U.S. norms and expectations, we used a data-driven, machine-learning approach that makes no assumptions about the underlying distribution of data. We used a clustering algorithm [16] to group infants based on times they were in the gahvora across the 24 hours. The algorithm converges on an optimal clustering without predefining the number of groups and without forcing individuals into groups if they do not clearly fit a group; see S1 File. For infants who failed to be grouped, we determined membership based on visual inspection of their timelines and accumulated hours in the gahvora.

## Results

Although we did not screen families based on gahvora use, 182 of 185 mothers used the cradle. Most mothers (89%) used the gahvora within the first two weeks of giving birth; 11% of mothers started 1–2 months after; and one infant was placed in the gahvora at 5 months when the family moved from Russia back to Tajikistan. Girls and boys started gahvora use at similar ages (*p* = .75).

### Gahvora cradle and associated materials

**Fig 2:**
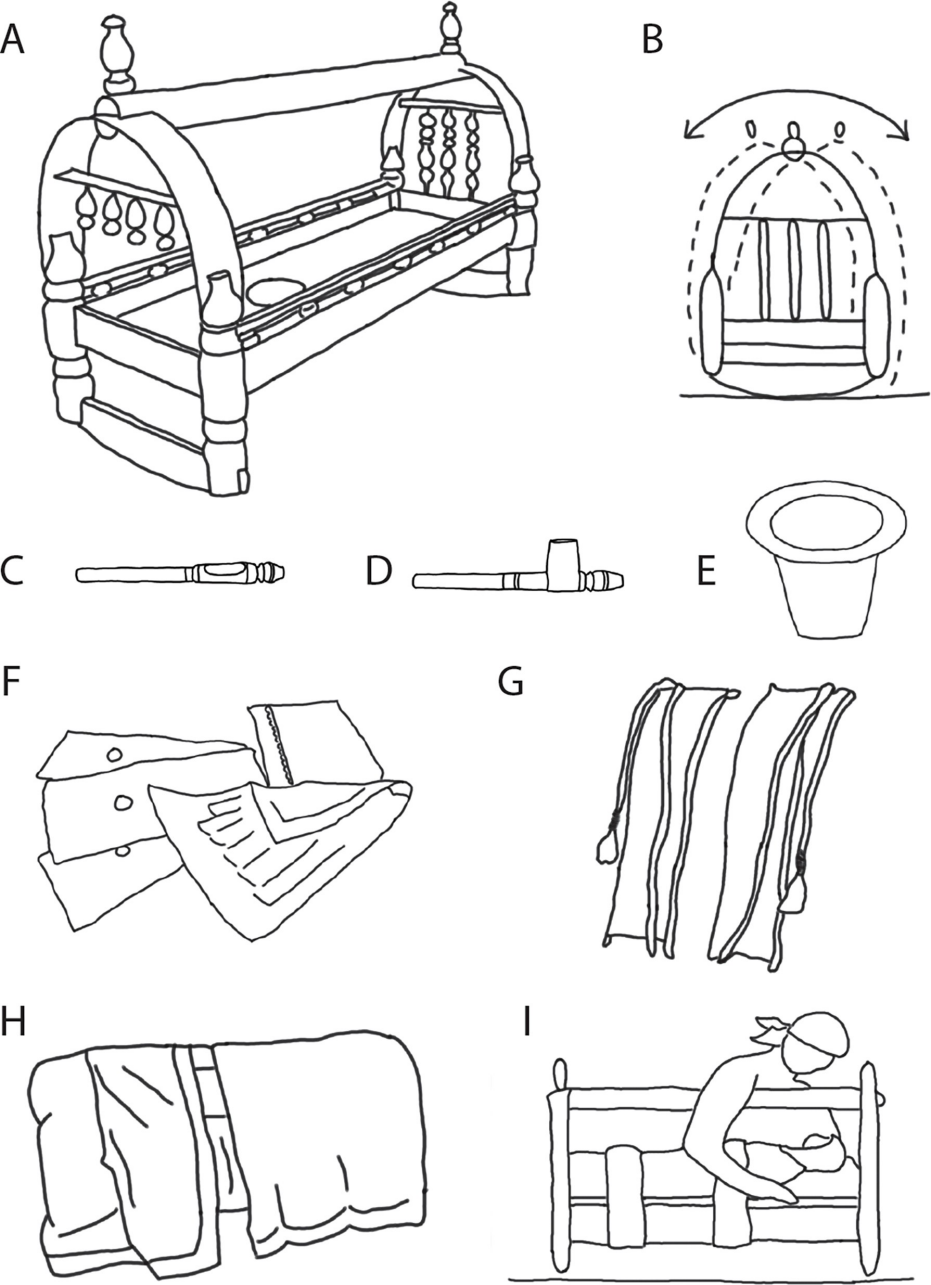
Elements of the “gahvora” cradle. (A) Side view. Traditionally, the cradle is handmade in walnut wood. Modern versions are machine-made from pine. (B) Front view illustrating curved base and how the cradle can be rocked from side to side. (C) External catheter called a “sumak” (note concave opening) for girls, and (D) for boys (note protuberance that fits around the penis) to collect urine. (E) A small bowl called a “tuvak” is placed under a hole in the bottom of the cradle to collect fecal waste and urine from the catheters. (F) Three mattresses, stuffed with millet or cotton, have holes to drain waste through the cradle bed into the tuvak. (G) Two wide bindings, made from cotton and velour, used to straighten and bind legs, arms, and torso, have long strings, which tie to the handle above the cradle. (H) Thick drapes (in winter) shut out light and keep infants warm; translucent drapes filter out patterned light (in summer) and protect infants from bugs; drapes cover the top and/or bottom halves of the cradle. (I) Mothers can breastfeed infants without removing infants from the cradle.

Mothers reported receiving the gahvora as a wedding gift. Some gahvoras had intricately detailed woodwork and others were plain, perhaps reflecting the family’s status and resources (Fig 1). Gahvoras (Fig 2A) were about the length of a 27- to 30-month-old (90 cm), narrow, and shallow (30 cm wide, 16 cm deep). They were made of wood, sat on a curved base for rocking (Fig 2B), and had a handle for carrying. An external “sumak” catheter specially devised for girls (Fig 2C) or boys (Fig 2D) siphoned urine into a “tuvak” bowl situated under a hole in the bottom of the cradle; the bowl collected feces directly (Fig 2E). Waste drained through holes in 3–4 mattresses (rotated for cleaning)—stuffed with cotton, straw, wool, or millet (Fig 2F), and topped with a soft pillow. The mattresses lined the shallow cradle bed, which had no side rails. To prevent infants from falling out, cotton swaddling cloth and wide bindings of cotton and velour (Fig 2G) wrapped around the baby and attached to the handle. Some caregivers hung pacifiers or small, dangling toys from the handle. Drapes covered the top and bottom of the cradle to block light, keep infants warm, and protect infants from flying insects and other environmental elements (Fig 2H). Some gahvora bindings and drapes were vibrantly colored with elaborate patterns, decorated with sequins and other trimmings; others were simple straps, blankets, and sheets. Mothers breast- or bottle-fed while infants were in the cradle (Fig 2I).

### Restrictive and expedient components of the cradling practice

**Fig 3:**
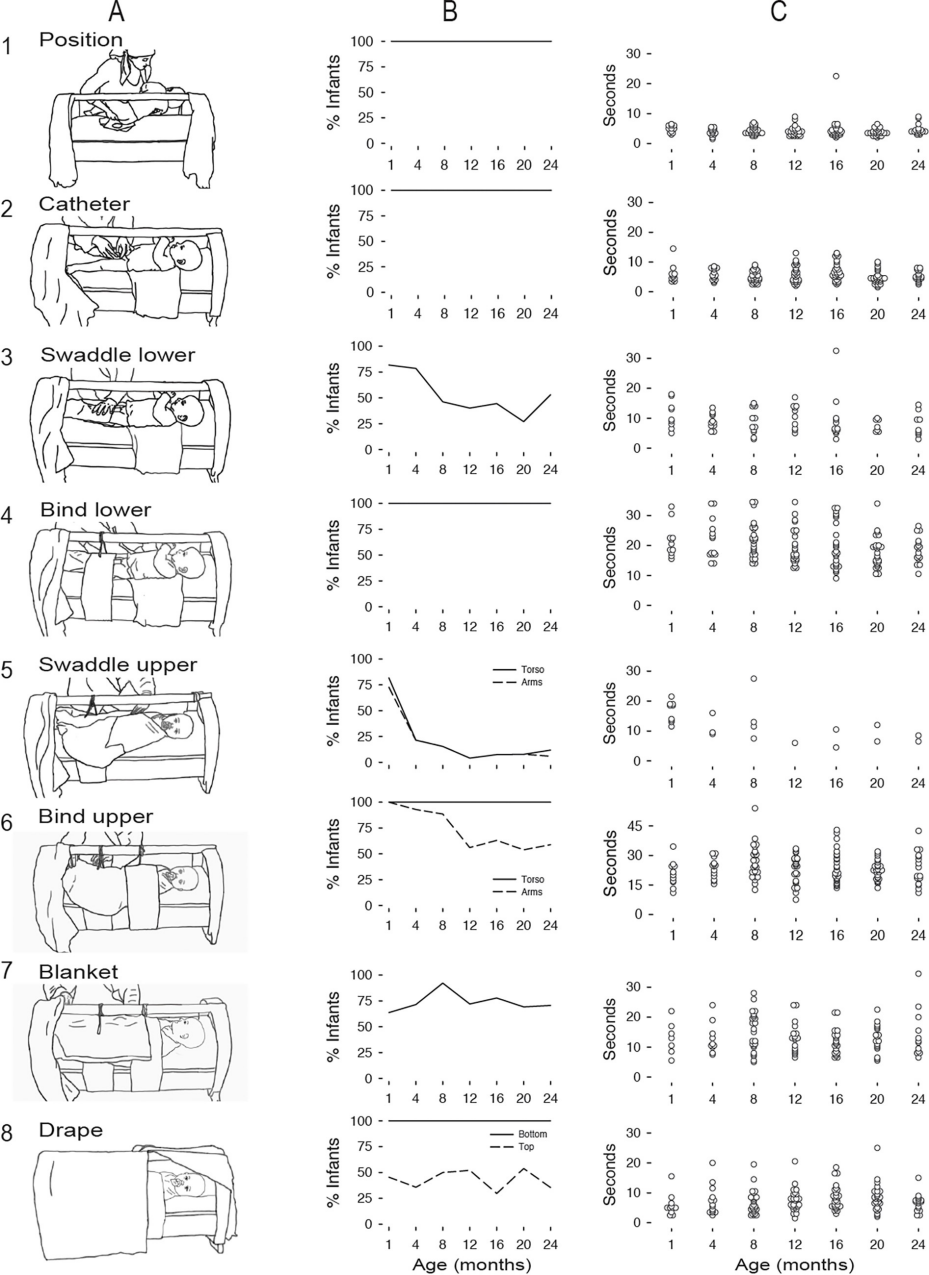
Quantitative description of the cradling process. (A) We identified 8 sequential steps. (B) Percentage of dyads displaying each step across age. Solid lines show steps that were universal across dyads; dashed lines show steps that only some mothers displayed. (C) Duration (in seconds) of each step for each dyad across infant age groups. Symbols denote individual data from each dyad.

Cradling resulted in severe restriction of infants’ limbs, torso, and vision. We identified 8 cradling components (Fig 3A): (1) *positioning* infants supine with buttocks over the hole in the mattress; (2) *catheterizing*; (3) *swaddling* the legs (4) *binding* the legs; (5) *swaddling* the arms; (6) *binding* the arms and/or torso; (7) *blanketing*; and finally (8) *draping*. All babies wore short- or long-sleeved t-shirts under the wrappings; 72% of mothers removed infants’ pants; the other mothers kept infants’ pants on, but pulled them down to infants’ thighs, presumably for convenience, creating additional restriction of the legs.

Some components were omnipresent across mothers and others were limited to a subset of mothers (Fig 3B). All mothers positioned infants supine directly over the hole with little repositioning; they catheterized babies by pulling the catheter out of the mattress hole and securing it in place while tightly gripping infants’ legs; they bound babies’ legs and torsos with straps and knotted the strings around the gahvora handle several times to prevent release; and they placed a drape over infants’ feet (Fig 3B components 1, 2, 4, and solid lines in 6 and 8). Swaddling was confined to a subset of mothers: 48% swaddled infants’ legs and 14% swaddled their arms. Mothers sometimes swaddled younger infants’ heads so that the head could not turn from side-to-side or nod up and down. Mothers (70%) bound infants’ arms by straightening them alongside infants’ torsos (dashed line in component 6, Fig 3B). Blanketing (75%) and draping infants’ faces (43%) were limited to some mothers. Swaddling legs, swaddling arms and torso, and binding the arms, decreased with infants’ age, χ^2^ 6, *N* = 146) > 15.9, all *p*s < .01 (Fig 3B components 3, 5, and dashed line in 6). Blanketing (with 1–2 blankets) and draping the cradle (with 1–4 coverings) did not change with infants’ age (Fig 3B components 7 and dashed line in 8).

Mothers’ rapid and consistent use of materials was striking, even to casual observation. Across mothers, each component took mere seconds to complete (Fig 3C). Positioning (*M* = 4.09 s, *SD* = 2.08) and catheterizing (*M* = 5.46 s, *SD* = 2.53) were particularly fast with low variability. Time to swaddle the legs (*M* = 9.11 s, *SD* = 4.71) and arms (*M* = 12.70, *SD* = 5.72), blanketing (*M* = 13.11 s, *SD* = 5.67), and draping (*M* = 7.18 s, *SD* = 4.06) did not differ in duration, *p* > .10, and were highly variable because 64% of mothers used multiples of these materials. In colder months, mothers used more swaddles, blankets, and drapes (and blankets and drapes were thicker and heavier) than in warmer months, when drapes were translucent to protect infants from bugs. Binding the legs (*M* = 19.74 s, *SD* = 6.16) and arms (*M* = 23.34, *SD* = 7.40) were most time-consuming: The bindings had to encircle the entire cradle before mothers tied a knot on the handle, and mothers had to smooth the bindings to avoid added friction on infants’ skin. A 7 (age) x 8 (components) mixed measures ANOVA on duration confirmed a main effect for components, *F* (7, 70) = 25.31, *p* < .001, but no effect for age or interaction.

### Consistent sequence of components

**Fig 4:**
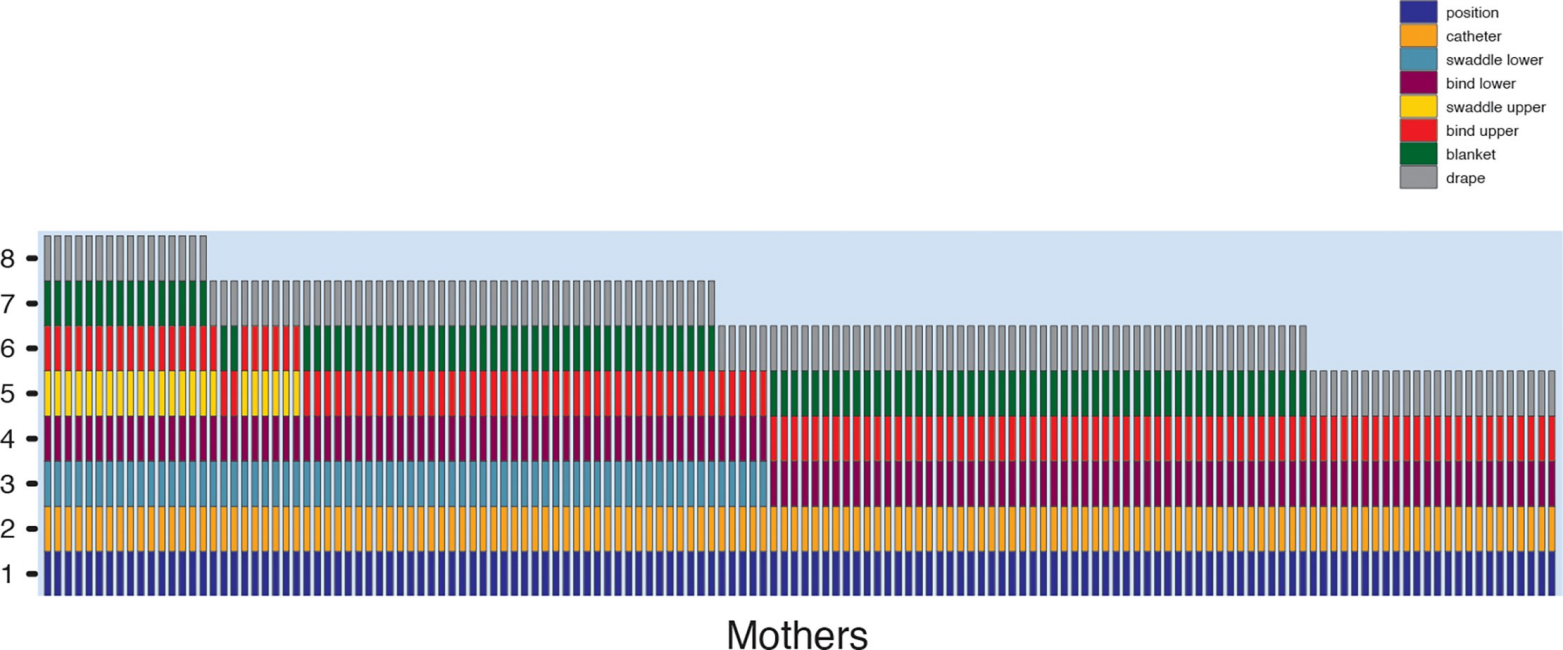
Distribution of cradling steps executed in order (read from bottom to top) for 146 mothers. Each bar represents one mother. Data are sorted based on mothers who completed all 8 steps to mothers who completed only 5 steps.

Fig 4 shows each component in the order displayed by each mother (component 1, positioning, is at the bottom, and component 8, draping, is at the top). Regardless of the total number of components, the order was uniform. In the figure, mothers are arranged, from left to right, from those who displayed the most to fewest components. Of the 11% of mothers who used all 8 components, most (68.7%) were mothers of younger infants (newborn, 4-, and 8-month-olds), χ^2^ (6, *N* = 146) = 18.95, *p* < .01.

#### Acquiescent infants

Infants were calm throughout the cradling process. Only 7.5% (*n* = 11) displayed any negativity (fussing or fidgeting), and they fussed across multiple components.

#### Feeding and rocking

Many mothers (40%) spontaneously breastfed infants while infants were bound in the cradle. Bouts of breastfeeding lasted *M* = 5.35 minutes, and as long as 25 minutes. A few mothers (11%) offered a bottle to their infants in the cradle. Most mothers (83%) spontaneously rocked the gahvora, with bouts lasting *M* = 4.37 minutes, and as long as 22 minutes. Most mothers (77%) displayed 1 to 2 bouts of prolonged, vigorous rocking, in which they tilted the cradle to its maximum attainable angle from the rest position by actively pushing and pulling the cradle rather than relying on gravity to move it back and forth.

### Gahvora use over the day and across development

**Fig 5:**
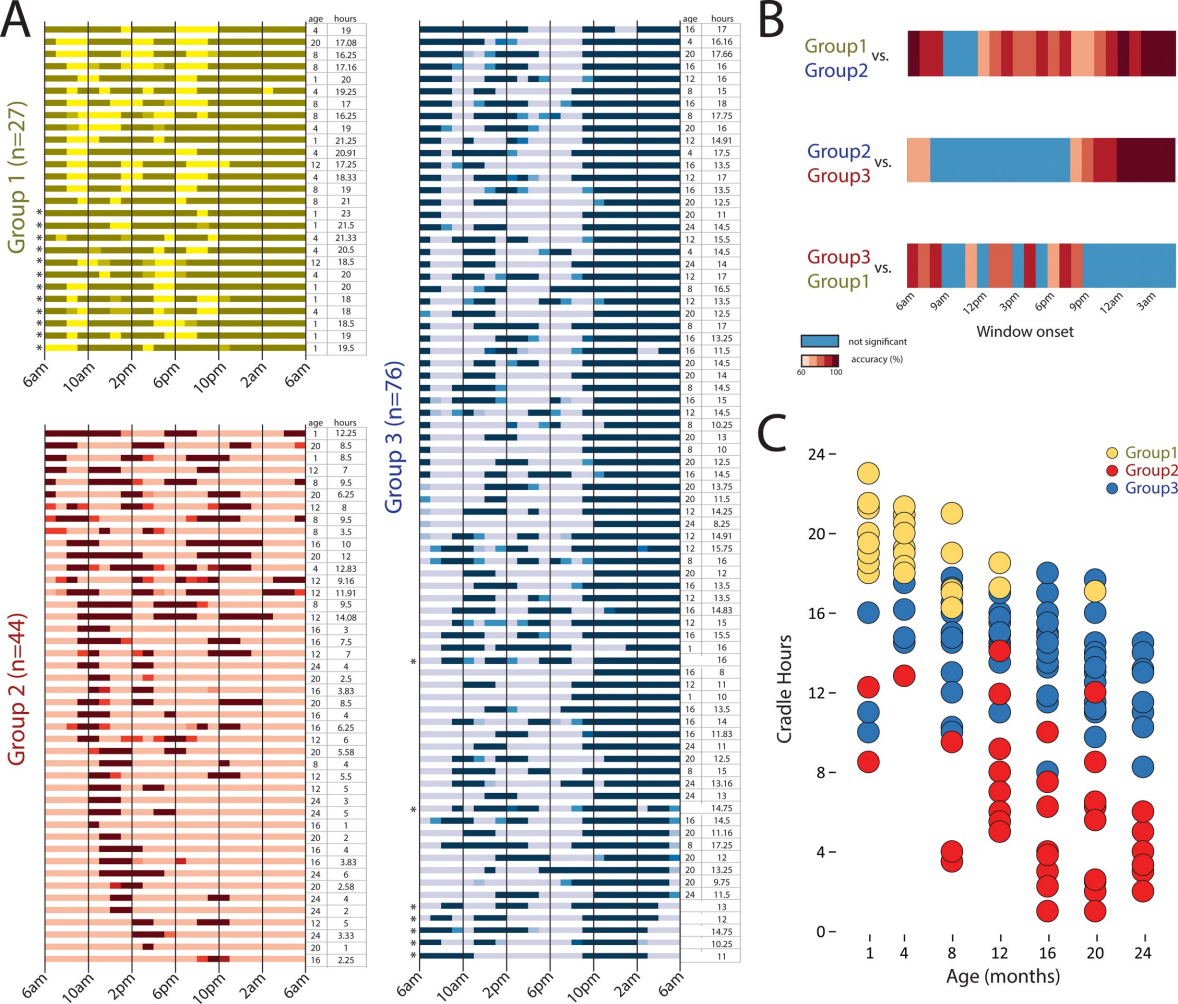
(A) Cluster classification based on distribution of gahvora hours during a 24-hour day using time-diary data. Timelines for each infant (represented by the horizontal raster bars), from 6 a.m. on the previous day until 6 a.m. on test day, split by cluster group. Dark colored bars represent times when infants are in the gahvora, light-colored bars show times when infants are out of the gahvora. Medium-shaded bars indicate that infants were in the gahvora for a fraction of the hour. (B) Show hour-block increments that best discriminate the cluster groups. Dark to medium red indicate 80–100% accuracy discrimination. Blue regions show hours that do not distinguish groups. (C) Accumulated cradling during the 24 hours across the 7 age groups. Symbols represent individual infants.

Families varied dramatically in the amount and distribution of gahvora use. The machine-learning algorithm clustered 128 of the 147 infants with time diaries into 3 groups. We manually assigned the remaining 19 infants who were not clustered (12 into group 1 and 7 into group 3). The group raster plots in Fig 5A show each infant’s timeline from 6 a.m. on the previous day to 6 a.m. on the test day (including the manually classified infants, marked by asterisks). Dark-shaded horizontal bars represent times infants were in the gahvora for a full hour; medium-shaded bars represent times infants were in the gahvora for part of an hour; and light-shaded bars represent times when infants were outside the gahvora.

The 27 infants in Group 1 (yellow bars) spent long periods in the gahvora, accumulating *M* = 19.13 hours (*SD* = 1.72), distributed across the 24 hours. Indeed, 10 Group 1 infants spent ≥ 20 hours in the cradle. For the 44 infants in Group 2 (orange bars) time in the gahvora occurred in short bursts, primarily between 10 a.m. and 10 p.m. (*M* = 6.09 hours, *SD* = 3.34). To our surprise, Group 2 infants were out of the gahvora at night. The 76 infants in Group 3 (blue bars) spent most of their time in the gahvora between 9 p.m. and 7 a.m., accumulating *M =* 13.82 hours/day (*SD* = 2.29).

Specific cradling hours distinguished among infants in the three groups. A machine-learning classification algorithm systematically compared Groups 1 to 2, Groups 2 to 3, and Groups 1 to 3 for each 2-hour time window (6 a.m. to 8 a.m., 7 a.m. to 9 a.m., etc.) to determine which windows discriminated between groups; see S1 File. The hued red regions in Fig 5B denote group differences with varying degrees of classification accuracy and the blue regions denote no differences between groups. Infants in Group 1 vs. 2 differed for all hours except 9 a.m. to 12 p.m. Group 1 vs. 3 showed similar gahvora use between 9 p.m. and 6 a.m. and four other times. Group 2 vs. 3 showed similar gahvora use between 8 a.m. and 8 p.m., but differed between 8 p.m. and 8 a.m. Manipulation of window durations—from the 2-hour time window to the full 24-hour day—converged on the same result; see S1 File.

Cradling hours decreased with infants’ age (Fig 5C), *r*(147) = -.54, *p* < .001. Most of Group 1 included the youngest infants (*M* = 4.74 months, *SD* = 4.84). Groups 2 and 3 were older, on average (*M*s = 15.27 and 14.31 months, *SD*s = 6.29 and 6.34, respectively), but included infants of most ages. Nonetheless, at each age, infants showed a wide range in cradling hours (e.g., 1-month-olds had 8.5–23 hours and 24-month-olds had 2–14.5 hours).

## Discussion

A few years ago, we stumbled across a report by Save the Children [1] on infants bound head to toe in a gahvora cradle. We set out to determine whether the report was true and began our foray into cultural research in Central Asia. Indeed, cradling severely restricted infants’ movements: Caregivers tightly swaddled and bound infants’ arms, legs, and torsos to the cradle bed. The catheter and hole in the cradle bed allowed caregivers to keep infants clean while cradled for long stretches of time. In most cases, infants could only move fingers and toes.

Mothers showed striking uniformity in cradling components, sequencing of components, and timing of each component. Time diaries showed large individual differences in duration and frequency of cradling across a 24-hour day, ranging from 30 minutes to 23 hours. Machine learning identified three patterns of gahvora use based on whether caregivers used the cradle during daytime hours, nighttime hours, or both. Younger infants spent more time in the cradle than older infants.

### Value of a cultural approach: Discoveries!

Our cultural venture revealed several important discoveries. In addition to the severe physical restrictions of cradling, we found that infants’ visual experiences may be restricted because the gahvora is often covered with heavy drapes in colder weather and translucent drapes in warmer weather. In the winter, up to four opaque drapes cover the gahvora, including the portion over infants’ face. The drapes limit exposure to sunlight, which may be a factor associated with vitamin D deficiency reported in infants in this region [17]. In the summer, translucent drapes allow light and shadows, but images are blurred. Even without the drapes, while strapped horizontally in the cradle, infants cannot benefit from a change in visual scenery (only the ceiling is potentially visible unless caregivers and siblings lean over the cradle to bring their face into infants’ view). Note, U.S. infants also spend large proportions of the day lying supine at young ages, and only see faces that others bring into infants’ view [18]. Thus, the visual experiences of infants in the gahvora differ from those of swaddled or cradle-boarded infants in other cultures who are belted vertically to caregivers’ backs with their faces exposed [3–4, 19].

We expected the practice of cradling to differ across mothers. After all, caregivers in Western cultures use different, idiosyncratic procedures to diaper, bathe, and soothe their infants, and put them to sleep. However, Tajik mothers cradled their infants in highly uniform ways, using the same materials in the same sequence with the same timing, regardless of mothers’ or infants’ ages, the region of the country, and the number of children in the family. Although some components—positioning infants and draping, for example—must occur in a particular order, binding infants’ arms, legs, and torso, and placing the external catheter could presumably vary in their ordering. To us, it seemed intuitive to restrict infants’ arms and torso before dealing with the catheter and legs to prevent infants from removing the catheter. In fact, the Save the Children report [1] stated that mothers cradled infants starting at the top of infants’ bodies, and worked their way down. Instead, no mother deviated from the bottom-up order.

We were surprised by how quickly and efficiently mothers cradled their infants, and their ability to multi-task while doing so. Watching mothers put their infants into the gahvora was like watching a routine medical procedure—mothers resembled a nonchalant, matter-of-fact medical professional operating on autopilot. Mothers often cradled their babies while carrying on conversations with several people in the room while barely glancing at their hands or infants. The speed and precision of executing each component was unlike any childrearing routine we have observed. Take diapering in the United States, for example. Mothers find many different solutions for diapering: They stand infants or lay them supine, offer toys as a distraction or chat with infants as they go, and choose disposable or cloth diapers. During diapering, infants often resist, fuss, or run away. In contrast to diapering, cradling seems more method than mayhem and babies remained placid.

Despite uniformity in the components and pace of cradling, mothers cradled infants at different times over the 24-hour day and for different durations. We expected longer durations of cradling for younger infants, which we saw. But many older infants (20% of 12- to 24-month-olds) were cradled for more than 15 hours per day. We also expected caregivers to reserve the gahvora for nighttime use and naps. Indeed, half of mothers (52%) kept their infants in the gahvora between 9 p.m. and 7 a.m., with intermittent use during daytime hours. However, to our surprise, 30% of mothers limited gahvora use to daytime hours and rarely used it during the night. Instead, infants slept unswaddled on kurpacha mats on the floor alongside their caregivers. Co-sleeping is common in many cultures, offering the convenience of breastfeeding and perhaps rendering protection against SIDS [20]. The daytime gahvora users included infants across age groups. The remaining infants (18%) were in the cradle during both daytime and nighttime hours. This group included younger infants. Thus, the gahvora could function like a crib, a playpen or baby minder, and a potty.

Despite spending long periods of time in the cradle, infants are entrenched in family life. Typically, restrictive environments conjure up images of orphanages where infants endure extreme neglect, are tied to their cribs, are fed with bottles propped on a pillow, and have rare opportunities to interact with the few available caregivers [21–23]. In contrast, Tajik caregivers responded immediately to vocalizations from their cradled infants by feeding them, rocking them, or singing to them. Mothers breastfed their infants by kneeling over one side of the gahvora then switching to the other side. In contrast to orphanages in which children far outnumber caregivers, in Tajikistan, mothers, grandmothers, aunts, neighbors, and older siblings were available, interchangeable, and responsive. We observed Tajik children of all ages, including siblings and village children, surrounding the gahvora and interacting with the target infant. In Tajik families, children are prized and the center of family life. Gahvoras are treasured gifts passed down for generations.

Caregivers commonly rocked the gahvora with intense vigor that appeared “rough” to our Western eye. Although we did not quantify the amplitude of rocking movements, we frequently observed high-amplitude rocking on video. Perhaps rocking appeared rough because Western caregivers are accustomed to handling “fragile” babies with extreme care [2]. However, caregivers in some African and Caribbean cultures consider rough handling to be integral to childrearing. Caregivers toss infants in the air and catch them, shake babies, and suspend them by the arms, ankles, and head as part of daily bathing and exercise routines [24–25].

Infants rarely protested being placed in the gahvora. They remained calm as their mothers bound and catheterized them. Anecdotally, mothers reported that their infants love to be put in the gahvora. Perhaps the gahvora soothes infants and instills a sense of calm, just as carrying and swaddling does in other cultures [26]. In the rare instances when infants fussed or cried, caregivers quickly responded by rocking the cradle, singing and talking to their infants, and uncovering the drapes to determine whether infants were hungry or uncomfortable.

### Methodological and conceptual challenges in obtaining cross-cultural data

Our research adventure in Central Asia reaped tremendous returns but posed numerous challenges. Tajikistan is a country historically closed off to scientific research [27] and potentially wary of Western researchers. Over multiple years, we cultivated relationships with international and government agencies, built interest in the topic of infant development, and established channels for disseminating information among local agencies. We were committed to capturing the richness of gahvora use with minimal bias and maximum transparency—by video recording cradling and obtaining detailed time diaries of the practice. But, the country’s developing infrastructure, limited internet, and unreliable parcel and mail services posed enormous challenges to the transfer of data. We used all available alternatives—including splitting files and using couriers—to transfer video data to our labs soon after data were collected. Language and cultural barriers presented another issue. Training was conducted in Russian; interviews were conducted in Tajik; and online coding was conducted in English and Russian. Maintaining integrity of the study protocol required extensive quality assurance efforts to prevent drift in data collection and misinterpretation of findings. To mitigate these problems, we stayed in constant contact with the Tajik research team (twice a week, on average) despite the 9-hour time difference, and sought corroboration across multiple Tajik collaborators to clarify questionable data entries.

An important challenge of cultural research is to recognize how best to interpret findings particularly because studies focused on children’s experiences and cultural practices in everyday settings are rare [28]. Rather than assume the routine of gahvora cradling in children’s everyday lives, we measured its prevalence. And we found that, gahvora cradling is pervasive in Tajikistan. Although we did not specifically recruit families based on gahvora use, nearly every mother reported having used a gahvora. But, why? Perhaps there are functional benefits of gahvora cradling given that the practice has endured for hundreds of years and has been observed in other neighboring regions of Central Asia and the Caucasus [29–31]. Similar to swaddling, cradling may induce and improve infants’ quality of sleep [32–33]. The environment in rural Tajikistan is potentially hazardous for infants—farm animals, farm tools, open fires, dangerous terrain such as pools of water and cliffs are in the immediate vicinity. Being bound in the cradle protects infants from such hazards. Similar to a Western playpen, the gahvora can be used to contain infants when caregivers need to complete tasks or leave the room. As a toileting method, the gahvora keeps infants clean and dry in an area with limited access to diapers and clean water. The gahvora shields infants from harsh winters in a country where homes are not insulated and electricity is limited.

### Remaining questions and ongoing work

Does the severe restriction of the gahvora affect infant development? Research in Western cultures suggests that excessive time spent supine causes an increase in brachycephaly (flattened back of the skull), plagiocephaly (flattened side of the head), and torticollis (weakened neck muscles on one side of the head) [34–38, 8]. But, there is no experimental evidence that a flattened head harms infant development [9, 39]. Moreover, therapies aimed to reshape the head appear to be no more effective than letting the condition work itself out [40].

Effects of restricted movement are not well known because research on infant development around the world is limited [2]. Even the World Health Organization—which published standards of motor achievement that infants should acquire by certain ages—excluded all of Central Asia and ignored culturally-specific childrearing practices [41]. Although we did not examine the consequences of gahvora use, cradled infants in Tajikistan and other parts of Central Asia learn to walk, talk, play with others, and participate in the routines of their community. It is possible that despite severe restrictions to movement while in the gahvora, infants have sufficient opportunities to move and explore when outside the gahvora and at later points in development. Our ongoing research aims to understand whether variation in time spent in the gahvora relates to infants’ developing motor and social skills.

### Conclusions

The value of a cultural approach is the discovery of new phenomena that challenge widespread assumptions about childrearing practices and the “natural” course of child development. Cultural beliefs, customs, and practices, geography, climate, and village resources compel caregivers to find ways to keep their children healthy and safe. Gahvora cradling is a widespread cultural practice throughout Tajikistan and presumably other parts of Central Asia. Yet, the practice flies in the face of Western norms, theories, and even WHO standards. To fully appreciate the enormous variability in children’s experiences, learning, and development, researchers must venture beyond the comfort of their labs to discover childrearing practices around the globe.

To convince researchers of the described phenomenon and inspire questions beyond the scope of the original study, these rare data, including nearly 400 hours of video, are stored in the Databrary (databrary.org) video library. These data are shared for the benefit of the broader scientific community and researchers with similar interests or different viewpoints.

## Supporting information

S1 File Contains information on the clustering and classification procedures. (DOCX)

S2 File Spreadsheet containing infants’ time diaries with hourly account of infants’ location, in or out of the gahvora. (CSV)

S3 File Spreadsheet containing the timing of each cradling component for each mother. (CSV)
