# Supplementary material for: The ties that bind: Cradling in Tajikistan
Source: PLoS One. 2018 Oct 31;13(10):e0204428. doi: 10.1371/journal.pone.0204428 (PMC6209138; doi:10.1371/journal.pone.0204428)
Supplement: S1 File — (DOCX) [file pone.0204428.s001.docx]

**Supplemental Materials**

**Clustering procedure**

To group the infants based on their cradling hours, we first calculated the similarity in distribution of cradling times across the 24-hours between each pair of infants using Euclidean distance:

$$d_{i,j}=\sqrt{\sum_{k=1}^{24} (x_{ik}-x_{jk})}$$

where *i* and *j* signify two different infants, and $x$ is the proportion of time each of the infants was in the cradle during hour *k*. Thus, we created a 147×147 similarity matrix in which cell *i,j* is the “distance” between infant *i* and infant *j* based on cradling hours. If the distance between the infants is low, the similarity between them is high and vice versa. We then followed the “fast search and find of density peaks” (Rodriguez & Laio, 2014) clustering procedure to group the infants. With this method, we first identified several infants as “centers” and then classified the rest of the infants according to the “center” to whom they were most similar. A center was characterized by: (1) high density in the similarity matrix such that many other infants were relatively similar; (2) large distances from other infants with higher densities, meaning relatively low similarity to other centers.

The density coefficient ρ reflects the number of infants whose distance from infant *i* in the similarity matrix is lower than a certain pre-defined cutoff distance (Rodriguez & Laio, 2014). Importantly, this cutoff distance is calculated separately based on the potential entropy of the data field as shown in previous papers (Wang, Wang, & Li, 2015), and it does not require prior definition. The formal equation for the density coefficient is:

$$\rho_{i}=\sum_{j=1}^{m} {\chi(d}_{i,j}-d_{c})$$

where the function $\chi$ is defined such that $\chi$ = 1 if $d_{i,j}-d_{c}$<0 and $\chi$ = 0; otherwise $d_{c}$ represents the entropy-based cutoff distance.

The distance coefficient δ reflects the minimal distance between infant *i* and the next infant with the next higher density (Rodriguez & Laio, 2014). Formally, the formal equation for the distance coefficient is:

$$\delta_{i}=\min_{j:\rho_{j}>\rho_{i}} (d_{ij})$$

The infant with the highest density was assigned the maximum value. Therefore, we defined $\delta_{i}=max(d_{ij})$. We then multiplied the distance coefficient and the density coefficient to create a γ score for each infant. γ scores are high when the infant has large distance and high density. From the γ distribution of all infants, we defined outliers as γ values that are at least 3 standard deviations above the mean γ. The number of outliers determined the number of clusters and each outlier was defined as a center. Each infant was assigned to a group according to the center to whom the infant was most similar. If the distance of infant *i* from all the centers was higher than the cut-off distance, infant *i* failed to be grouped, and we determined membership based on visual inspection and accumulated cradling hours (19 infants described in Results).

**Classification procedure**

To identify specific cradling hours that distinguish among infants in the groups, we constructed a linear classifier to decode group membership from cradling hours. The classifier was provided with the group label and corresponding cradling patterns across 24 hours. We used a Matlab implementation of a binary support vector machine (SVM) classifier (Chang & Lin, 2011 http://www.csie. ntu.edu.tw/~cjlin/libsvm) to discriminate each pair of groups. We applied a least squares cost function (C=1).

For each group comparison, the following classification procedure was implemented: First, data from each group were collected; this results in a 24×*N* matrix of values for each group, where *N* is the minimal number of infants across both groups (if a group included more than *N* infants, we randomly chose *N* infants in each iteration). Next, we randomly chose one infant from each group to be used as a test set and the classifier was trained on the remaining two datasets of *N*-1 infants. During the test stage, the classifier assigned labels to the left-out data (the trials which it was not trained on) and its performance was assessed. Classification performance was assessed on the test set as the average performance level across 1000 permutations (‘leave-one-out’). Significance was assessed by comparing the accuracy levels to a null distribution (*p* < 0.05) obtained by shuffling the labels of the data (5000 shuffles) and performing the same classification procedure as on the original data.

Finally, we systematically changed the input to the classifier. Given that the minimum window size is 2 hours, we first used cradling time from 6 a.m. to 8 a.m. as input to the classifier. We then continued the classification process in 1-hour increments (i.e., classifying the groups based on cradling time from 7 a.m. to 8 a.m. in the following step and so on until 6 a.m.). Then, we also increased the window size in 1-hour increments, starting from 2 hours until 24 hours. This allowed us to determine hours in which the use of the cradle significantly differed across the groups. Classification results between each pair of groups when window size = 2 hours are shown in Figure 5B. We found similar results when using the other window sizes.

**References**

Chang, C. C., & Lin, C. J. (2011). LIBSVM: A library for support vector machines. *ACM Transactions on Intelligent Systems and Technology (TIST), 2*, 27.

Wang, S., Wang, D., & Li, Y. (2015). Comment on “Clustering by fast search and find of density peaks”. *arXiv preprint arXiv:1501.04267*.

Rodriguez, A., & Laio, A. (2014). Clustering by fast search and find of density peaks. *Science, 34*, 1492-1496.
